# A multi-center prospective randomized controlled trial (phase III) comparing the quality of life between laparoscopy-assisted distal gastrectomy and totally laparoscopic distal gastrectomy for gastric Cancer (study protocol)

**DOI:** 10.1186/s12885-019-5396-8

**Published:** 2019-03-07

**Authors:** Chang Min Lee, Ji Ho Park, Chang In Choi, Han Hong Lee, Jae-Seok Min, Ye Seob Jee, Oh. Jeong, Hyundong Chae, Sung Il Choi, Hua Huang, Sungsoo Park

**Affiliations:** 1 Department of Surgery, Korea University College of Medicine, Seoul, Korea; 2 Department of Surgery, Gyeongsang National University School of Medicine, Jinju, Korea; 3 Department of Surgery, Medical Research Institute, Pusan National Universtiy Hospital, Busan, Korea; 4 Department of Surgery, College of Medicine, The Catholic University of Korea, Seoul, Korea; 5 Department of Surgery, Dongnam Institute of Radiological and Medical Science, Busan, Korea; 6 Department of Surgery, Dankook University College of Medicine, Cheonan, Korea; 7 Department of Surgery, Chonnam National University Medical School, Gwangju, Korea; 8 Department of Surgery, Catholic University of Daegu School of Medicine, Daegu, Korea; 9 Department of Surgery, Kyung Hee University Hospital at Gang Dong, Seoul, Korea; 10 Department of Oncology, Shanghai Medical College, Fudan University, Shanghai, China; 11 Department of Surgery, Korea University Medical Center Anam Hospital, Inchon-ro 73, Seongbuk-gu, Seoul, 02841 Korea; 12 Department of Gastric Surgery, Fudan University Shanghai Cancer Center, No.270 Dong an Road, Shanghai, 200032 China

**Keywords:** Gastric cancer, Laparoscopy-assisted distal gastrectomy, Totally laparoscopic distal gastrectomy, Randomized controlled trial

## Abstract

**Background:**

KLASS (the Korean Laparoendoscopic Gastrointestinal Surgery Study) is a time-honored study group that has established laparoscopic surgery for gastrointestinal disease in Korea and has performed some important studies for the rationale of laparoscopic gastrointestinal surgery. A multi-center RCT (randomized controlled trial) to compare the quality of life (QOL) of patients undergoing totally laparoscopic distal gastrectomy (TLDG) and laparoscopy-assisted distal gastrectomy (LADG) for gastric cancer, named as KLASS 07, has been currently prepared in Korea.

**Methods:**

Patients diagnosed as gastric cancer, with clinical stage IA (T1N0M0) or IB (T1N1M0 / T2N0M0) according to the 7th edition of the Americal Joint Committee on Cancer System, were randomized to receive either TLDG or LADG. For surgical quality control, the surgeons participating in this trial had to have performed at least 50 gastrectomies and at least 30 gastrectomies annually (regardless of open or laparoscopic surgery for gastric cancer). The patients who are allocated to TLDG group undergo intracorporeal anastomosis and those who are assigned to LADG undergo extracorporeal anastomosis for gastrointestinal reconstruction.

**Discussion:**

Thirty-one surgeons from 26 institutions were engaged in this trial. The primary endpoint is 30-day morbidity, and secondary endpoint is QOL assessed by the questionnaire score. The KLASS 07 trial is the first multi-center RCT to investigate whether there are significant and quantifiable differences between the QOL of TLDG and LADG. The findings from this trial are expected to be the critical clues for designing the detailed procedures during laparoscopic surgery for gastric cancer.

**Trial registration:**

The protocol of KLASS 07 (CKLASS 01) was registered in http://register.clinicaltrials.gov as NCT03393182 (Date of registration: January 2nd, 2018.).

## Background

Since Kitano et al. [1] firstly reported laparoscopy-assisted distal gastrectomy (LADG) with lymphadenectomy in 1994, this procedure has been widely accepted as a treatment option for early gastric cancer. Especially in Korea, LADG with lymphadenectomy was recognized as a safe and effective treatment modality for early gastric cancer (EGC) in a multi-center trial.

For the last decade, Korean Laparoendoscopic Gastrointestinal Surgery Study (KLASS) group has performed a prospective randomized controlled trial to show the clinical effectiveness of laparoscopic gastrectomy for early gastric cancer (Registered in www.clinicaltrials.gov as NCT00452751, KLASS 01) [2]. The interim result of KLASS 01 showed no significant difference of postoperative morbidity and mortality between open and laparoscopic gastrectomy [3]. Furthermore, the long-term result of this trial finally revealed that LADG is not inferior than open procedure in terms of the oncologic safety [4]. Since 2012, KLASS group has also progressed a prospective randomized controlled trial to show the feasibility of laparoscopic gastrectomy for locally advanced gastric cancer as well as early gastric cancer (NCT01456598, KLASS 02). By April 2015, KLASS group has finished the actuall enrollment for KLASS 02, and therefore many gastric cancer surgeons are waiting for the results of this multi-center study.

While KLASS group has verified the safety and effectivity of laparoscopic gastrectomy with lymphadenectomy for EGC, laparoscopic surgical techniques have been developed for the feasibility of intracorporeal procedures. As a result, more recently, many surgeons have adopted totally laparoscopic distal gastrectomy (TLDG) rather than LADG, that has been introduced since the initiation of laparoscopic surgery for gastric cancer. During LADG, reconstruction procedure, by which gastrointestinal continuity is recovered, has been extracoporeally performed through mini-laparotomy [1]. Unlike this, during TLDG, reconstruction procedure is intracoporeally performed without mini-laparotomy.

According to the previous reports, TLDG shows several advantages over LADG. For example, anastomosis can be safely performed regardless of tumor location during TLDG [5], and wound problem is less frequent in TLDG than LADG [6]. In addition, anastomosis can be feasibly performed in the obese patients [7, 8]. However, we could not conclude that the quality of life (QOL) of patients undergoing TLDG is superior than LADG. At present, although the previous studies have compared the clinical outcomes between TLDG and LADG [9, 10], there has been few prospective randomized controlled trial to compare the quality of life (QOL) between the patients undergoing TLDG and LADG.

To evaluate how each laparoscopic surgery affect QOL of patients with gastric cancer, it is necessary to compare the satisfaction degree and postoperative QOL (scoring by questionnaire) between the patients undergoing TLDG and LADG through a multi-center randomized controlled trial. The Clinical Trial Review Committee of the KLASS Group approved the protocol on July, 2016 and the study was activated on January, 2018. This study is the first prospective, randomized, multi-center trial that comparing QOL of TLDG and LADG in Korea.

## Methods

### Objectives

The primary endpoint of this study is to assess whether TLDG is superior than LADG in terms of the short-term morbidity. The secondary research endpoint is to compare TLDG and LADG in terms of the quality of life (QOL).

### Design

The study has received Ethical Committee approval in each participating institute. The KLASS 07 trial is a multi-center randomized controlled trial (RCT) that is evaluating the superiority of TLDG over LADG in 442 patients with early gastric cancer who have been recruited in 26 institutes in Korea. In addition, another multi-center RCT using the same protocol as KLASS 07 is ongoing in China (CKLASS 01). The protocol of KLASS 07 (CKLASS 01) was registered in http://register.clinicaltrials.gov as NCT03393182.

### Study population

The patient inclusion criteria were as follows: (1) histologically proven gastric adenocarcinoma (by preoperative gastrofiberscopy), (2) age between 20 and 80 years old, (3) Eastern Cooperative Oncology Group (ECOG) performance status of 0 or 1 (4) clinical stage IA (T1N0M0) or IB (T1N1M0 / T2N0M0) according to the 7th edition of the Americal Joint Committee on Cancer System [11] (Clinical stage was determined based on the finding of gastrofiberscopy and abdominal computed tomography), and (5) scheduled for laparoscopic distal gastrectomy with D1+ or D2 lymphadenectomy, and possible for R0 surgery by this procedures (Lymphadenectomy is performed on the basis of the criteria of the Japanese Gastric Cancer Treatment Guidelines 2010 (ver. 3).). The patient exclusion criteria were the following: (1) received gastric surgery (i.e. gastrectomy or gastrojejunostomy), (2) adhesion due to the previous intra-abdominal surgery, (3) history of chemotherapy or radiotherapy in the last 5 years, (4) need for combined organ resection due to aggression of gastric cancer of other disease, (5) received surgeries due to the primary cancer of other organs (Patients whose skin basal cell carcinoma or in situ cervical cancer are completely cured are exceptions.), (6) vulnerable people who cannot communicate or are pregnant (or planning to be pregnant), and (7) currently participating or participated in other clinical trials in the last 6 months. All patients freely gave informed consent to participate in the study and can decide to withdraw from the study at any time.

### RADOMIZATION, allocation and data collection

**Fig. 1:**
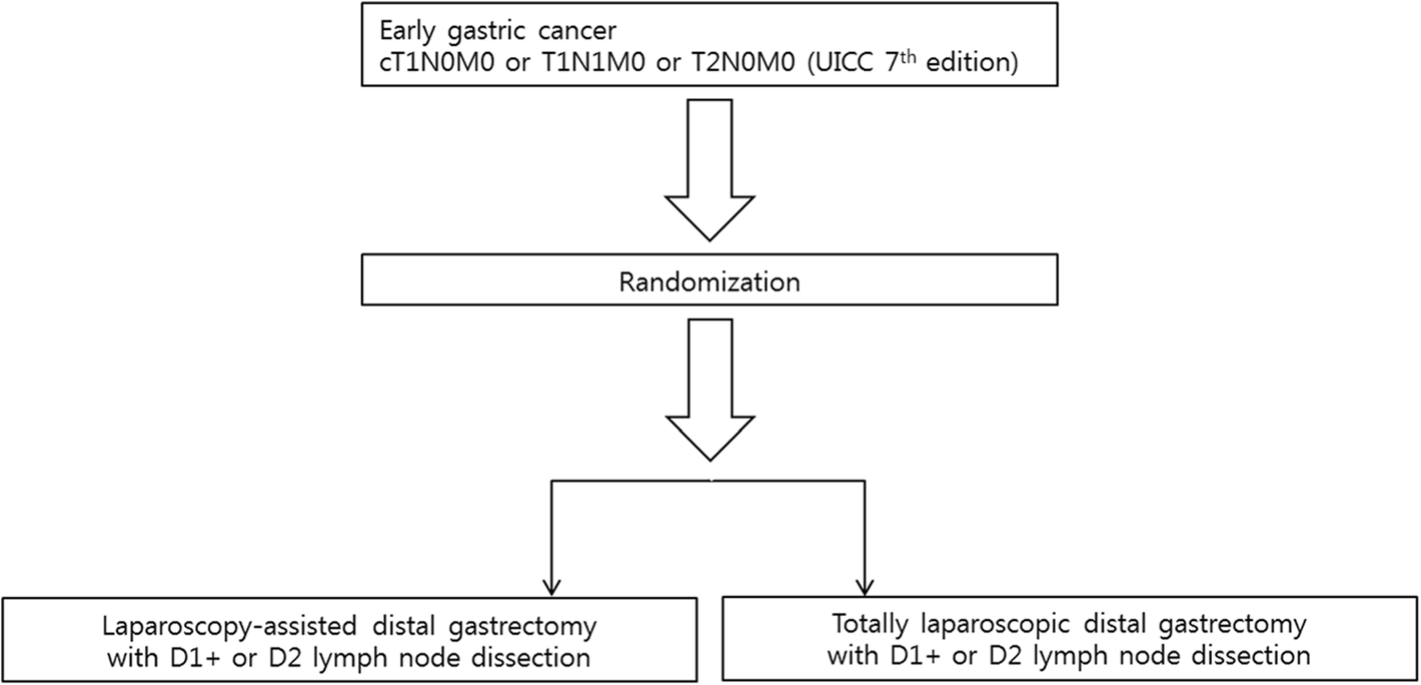
The scheme of study progress. Through the randomization, patients met the inclusion / exclusion criteria were assigned to either laparoscopy-assisted or totally laparoscopic distal gastrectomy group in 1 to 1 ratio

The scheme of study progress was showed in Fig. 1. After confirming the patients met the inclusion / exclusion criteria, each patient was registered in the trial by inputting the screening data including the age, sex, height, weight, body mass index (BMI), and ECOG score into the electronic clinical record form (available at www.klass07.com). The central data center then requested the preoperative data including ASA (American Society of Anesthesiologists physical status classification) score, associated disease, history of intra-abdominal surgery, and each score for the items of QOL questionnaire. After the preoperative data had been completed in the central data registry server, the enrolled patient underwent the randomization, which was coordinated by the central data center. Through the randomization, patients were assigned to either TLDG or LADG group in 1 to 1 ratio, and they are stratified according to the surgeons.

### Intervention strategies

For surgical quality control, surgeons could only participate in this trial if they had performed at least 50 gastrectomies, and over 30 gastrectomies annually.

A standard radical distal gastrectomy with D1+ or D2 lymphadenectomy was performed in both the TLDG and LADG groups. The extent of lymphadenectomy was determined on the basis of the criteria of the Japanese Gastric Cancer Treatment Guidelines 2010 (ver. 3). Omentectomy was performed partially and reconstruction was performed by the standard Billroth II (with or without Braun anastomosis) or Roux-en-Y or uncut Roux-en-Y fashion, depending on the preference of the surgeon.

The patients who are allocated to TLDG group undergo intracorporeal anastomosis for the recovery of gastrointestinal continuity after gastrectomy. The patients who are assigned to LADG group undergo extracorporeal anastomosis for gastrointestinal reconstruction.

### Main outcome parameters

The primary endpoint of this trial is the early postoperative morbidity, that is defined as complications that occur within 30 days after surgery [3]. Early postoperative morbidity is classified as follows: (1) wound morbidity: operation wound with seroma, hematoma, infection, dehiscence, or evisceration, etc.; (2) surgical site morbidity: anastomosis bleeding or leakage, duodenal stump leakage, postoperative bleeding, afferent loop or efferent loop obstruction, etc.; (3) lung morbidity: atelectasis, pleural effusion, empyema, pneumothorax, etc.; (4) intestinal obstruction morbidity: no return of bowel movement until 5 days after surgery, mechanical obstruction with an air–fluid level or paralytic ileus on simple X-ray, etc.; (5) urinary tract morbidity: frequency, nocturia, dysuria, increased white blood cell count on urine analysis, etc.; (6) intraabdominal abscess: the presence of septic fluid in the abdominal cavity that causes fever higher than 38 °C and is proven by abdominal sonography or computed tomography scanning; (7) postoperative pancreatitis: elevated serum amylase (> 150 U/L) with symptoms that are suggestive of pancreatitis such as back pain and fever; (8) pancreatic fistula: drain amylase content greater than 1000 U/L after postoperative day 3; (9) intestinal fistula: presence of a bowel to bowel or bowel to cutanous fistula tract that is confirmed by a fistulogram; (10) others: lymphorrhea, diarrhea, etc. The date of the morbidity diagnosis will be recorded.

The secondary end point is the questionnaire score regarding QOL. This is assessed by the Korean versions of the EORTC QLQ-C30 (version 3.0) and STO22 questionnaires [12]. The EORTC QLQ-C30 consists of a 30-item cancer-specific integrated system for assessing key functional aspects of health-related quality of life (QOL), the global QOL, and symptoms that commonly occur in cancer patients. It incorporates five function scales (physical, role, cognitive, emotional, and social), three symptom scales (fatigue, pain, and nausea and vomiting), a global health and QOL scale, and single items for assessing additional symptoms that are commonly reported by cancer patients (e.g. dyspnea, appetite loss, sleep disturbance, constipation, and diarrhea), and the perceived financial impact of the disease and treatment. Of the 30 items, 28 items are scored on a four-point Likert scale and the remaining two items for the global health status scale are scored on modified seven-point linear analog scales. All scales were linearly transformed to a 0 to 100 score, with 100 representing the best global health status or functional status or the worst symptom status. The EORTC QLQ-STO22, a stomach cancer-specific questionnaire, consists of 22 items. It includes five scales (dysphasia, eating restrictions, pain, reflux, and anxiety) and four single items (dry mouth, body image, taste problems, and hair loss) that reflect disease symptoms, treatment side effects, and emotional issues that are specific to gastric cancer, with high scores indicating worse symptomatic problems.

Postoperative QOL data are collected by directly contacting patients at baseline (before surgery) and on every follow-up visit after surgery (3, 6 and 12 months after surgery).

### Other outcome parameters

#### Short-term clinical outcomes (other than the morbidity)

We investigated the short-term clinical outcomes including the total operation time, time for reconstruction, hospital stay, estimated blood loss, and day of starting liquid or semi-blended diet. In addition, we also measured the pain score using Wong-Baker Faces pain rating scale at 24 h after the surgery is completed [13].

#### Late postoperative morbidity

Late postoperative morbidity is defined as complications that occur after postoperative day 30. Late postoperative morbidity is classified as follows: (1) adhesive ileus: mechanical or paralytic obstruction on CT scan accompanied by symptoms such as abdominal pain, vomiting, and no gas passing; (2) anastomosis stricture: narrowing of the anastomosis that is confirmed by upper gastrointestinal series; (3) reflux esophagitis: esophageal erosion to the stricture that is confirmed by endoscopy; (4) malnutrition: iron deficiency anemia, megaloblastic anemia, or steatorrhea; (5) dumping syndrome. The date of the morbidity diagnosis will be recorded.

#### Mortality

This item refers to all hospital deaths since the day of surgery. The date of death, if the patient died, will be recorded.

#### Cost-effectiveness

At the time of discharge, the total cost and surgery-related cost will be calculated and the two groups will be compared with regard to cost-effectiveness. Operation cost and other fees during the hospital stay, and the number of used automatic suture machine cartridge during the surgery.

#### Postoperative gastrofiberscopic findings

After 6 and 12 months from the operation, the gross findings on gastrofiberscopy (food retention, gastritis, bile reflux) are evaluated based on RGB classification [14].

### Follow-up

The patients in both groups will be followed up 25 ± 7 days and 3, 6 and 12 months after surgery. At every follow-up, a physical examination will be performed. A complete blood count, nutritional indicators (total protein and albumin), and liver-function testing will be performed 25 ± 7 day and 6 and 12 months after surgery. Endoscopy will be performed twice a year (6 and 12 months after surgery).

### Sample size calculation

This trial is designed to compare TLDG and LADG in terms of the short-term morbidity and QOL. According to interim results of the multi-institutional prospective study (KLASS 01) that is reported in 2010, the complication rate after the LADG was 11.6% [3], while it was 16.4% according to another prospective single-institutional study reported in 2015 [15]. In addition, according to the final outcomes of KLASS 01 study, the incidence of postoperative complication was 13% [16]. Estimated shor-term complication rate for LADG based on the previous results mentioned above is 13.6%. On the other hand, according to the recent studies on short-term clinical outcomes of TLDG [10, 15, 17], the short-term complication rate of TLDG was expected to be 7.5%. Therefore, the two surgical methods might show the morbidity differed by 6.1%. The hypothesis to be tested is that the 30-day morbidity rate of TLDG is 6.1% less than that of LADG. Allowing for a dropout rate of 10%, the planned sample size was 442, with 221 cases per each arm. This will provide a power of 80% to reject the null hypothesis with a significance level at less than 0.05.

### Study monitoring and interim analysis

A Trial Steering Committee meets every 6 months and will be responsible for drafting the final report and submission for publication. The monitoring reports are submitted to and reviewed by the data and safety monitoring committee every 6 months. Soon after recruitment had started, a data and safety monitoring committee met at the start of the trial to establish a charter, and will continue to meet at least annually to determine if there are any ethical problems.

To evaluate the safety of this trial, one interim analysis was planned. The interim analysis tested the hypothesis that the TLDG- and LADG-associated morbidity in this trial did not differ significantly from the morbidity reported in previous studies on open gastric cancer surgeries.

### Progress

The actual enrollment will be ended at the time when the planned sample size is achieved.

### Participating institutions (arranged alphabetically)

Ajou University Hospital, Asan Medical Center, Seoul St. Mary’s Hospital, Daejeon St. Mary’s Hospital, Incheon St. Mary’s Hospital, Uijeongbu St. Mary’s Hospital, Chonbuk National University Hospital, Chonnam National University Hospital, Chonnam National University Hwasun Hospital, Daegu Catholic University Medical Center, Eulji University Hospital, Dankook University Hospital, Dongnam Institute of Radiological and Medical Science, Inje University Busan Paik Hospital, Inje University Haeundae Paik Hospital, Jeju National University Hospital, Chosun University Hospital, Keimyung University Dongsan Medical Center, Konyang University Hospital, Korea University Medical Center Anam Hospital, Korea University Medical Center Ansan Hospital, Korea University Medical Center Guro Hospital, Kyung Hee University Hospital at Gangdong, Pusan National Universtiy Hospital, Pusan National University Yangsan Hospital.

## Discussion

As we described above, we named this study as KLASS 07. KLASS is a time-honoured study group that has established laparoscopic surgery for gastrointestinal disease in Korea, and has performed some important RCTs for the rationale of laparoscopic gastrointestinal surgery. In 2016, KLASS group admitted the launching of KLASS 07 study, since they agreed with the necessity of RCT to compare the QOL between LADG and TLDG.

However, even though it is general to define the primary endpoint as QOL score, the short-term morbidity was designated as the primary endpoint of KLASS 07. There are 2 representative reason for this: i) First, regarding this subject, there has been few comparative result about QOL, and therefore we could not find any reference for sample size calculation. ii) Second, in the most of previous studies, the score of QOL questionnaire is not so different between the patients. Thus, it is difficult to provide the difference regarding superiority of non-inferiority, by which investigators find the rationale of sample size. Here, from our clinical experience, we expect that short-term morbidities have a strong effect on QOL of patients who underwent surgery, regardless of the type of surgery. With these reasons in mind, we defined the primary endpoint as “30-day morbidity”.

Although Woo et al. [15] previously reported a result of RCT regarding this topic, KLASS 07 embeds some discriminating values due to the following reasons.

First, it is necessary to perform multi-center RCT to compare the QOL between TLDG and LADG. To contemplate the superiority of one side or equality between both sides, we should get the universality of the surgical performance. It is not rational to conclude with the result of single institute, because one surgeon’s experiences do not representate the clinical outcomes of laparoscopic gastrectomy performed by many gastrointestinal surgeons. In addition, according to the current recommendation regarding the clinical evidence the previously performed single center RCT can be classified as ‘small RCTs with unclear results’, and deserves to level II evidence [18]. To acquire a level I evidence, we should perform a multi-center RCT [18]. The steering committee of KLASS 07 has already recruited 31 surgeons of 26 institutes.

Another value of KLASS 07 is also correlated with the universality. Regarding this comparison between LADG and TLDG, it is not sufficient to conclude with the limited number of patients. Woo et al. [15] involved only 55 patients in each arm. This sample size was defined based on one retrospective study (Tanimura et al. [19]’ study). In order to acquire the reliable basis for sample size calculation, we referred to 5 literatures (3 prospective studies and 2 retrospective studies), including the interim and final results of KLASS 01 study.

Furthermore, other than the QOL outcomes, KLASS-07 study additionally deals with the endoscopic findings and cost issues. The endoscopic findings will be investigated based on RGB classification, in which the food residue, gastritis, and bile reflux were systematically investigated. This subjects are known to be deeply related with QOL of patients, and therefore it should be investigated by an multi-institute RCT. In addition, our protocol suggests the diverse types of reconstructions, including Billroth II (with or without Braun anastomosis), Roux-en-Y gastrojejunostomy, and uncut Roux-en-Y gastrojejunostomy. Therefore, we can compare these reconstruction methods in terms of the clinical outcomes and QOL, acquire the information regarding the trend of reconstruction. Although the several literatures showed the lower incidence of gastritis and bile reflux in Roux-en-Y reconstruction than Billroth II [20–23], there has been no multi-center RCT regarding this issue. In addition, most of these studies did not explain the role of Braun anastomosis in patients underwent Billroth II reconstruction during laparoscopic gastrectomy. Moreover, as KLASS-07 study deals with laparoscopic procedure, in which postoperative adhesion less caused, we planned to analyze the relationship between the reconstruction method and internal hernia.

In addition, regarding the reconstruction method, KLASS-07 study protocol reflects the current status of gastric cancer surgery. Unlike the 2000 s when KLASS-01 study was ongoing, surgeons’ preferences to the reconstruction methods have changed after introducing TLDG. This trend is the reason why we exclude Billroth I reconstruction from KLASS-07 study. According to the results of Korean Gastric Cancer Association Nationwide survey in 2014, Billroth I reconstruction was less adopted in TLDG (35.1%) than LADG (66.9%) [24]. In other words, since TLDG was introduced, gastrojejunostomy-based reconstructions (i.e. Billroth II, Roux-en-Y gastrojejunostomy, and uncut Roux-en-Y gastrojejunostomy) have increased and Billroth I have decreased. With these reasons, Billroth I reconstruction was regarded to be a confounder in RCT comparing TLDG and LADG, although we could not confirm the obvious reason of avoiding Billroth I during TLDG.

## Conclusions

This multi-center RCT is still required to determine whether there are significant and quantifiable differences between the QOL of TLDG and LADG. The KLASS 07 trial is the first multi-center RCT regarding this topic. The findings from this trial are expected to be the critical clues for designing the detailed procedures during laparoscopic surgery for gastric cancer.

## Abbreviations

ASA: American Society of Anesthesiologists physical status classification
BMI: Body mass index
ECOG: Eastern Cooperative Oncology Group
EGC: Early gastric cancer
EORTC: European Organisation for Research and Treatment of Cancer
KLASS: The Korean Laparoendoscopic Gastrointestinal Surgery Study group
LADG: Laparoscopy-assisted distal gastrectomy
QLQ-C30: Quality of Life Questionnaire – Core Questionnaire 30
QOL: Quality of life
RCT: Randomized controlled trial
RGB: Residue, Gastritis, and Bile
STO22: The Stomach 22 module
TLDG: Totally laparoscopic distal gastrectomy
